# Wettability of amorphous and nanocrystalline Fe_78_B_13_Si_9 _substrates by molten Sn and Bi

**DOI:** 10.1186/1556-276X-6-318

**Published:** 2011-04-08

**Authors:** Ping Shen, JianXin Sun, Jun Yang, Yan Qi, QiChuan Jiang

**Affiliations:** 1 Key Laboratory of Automobile Materials, College of Materials Science and Engineering, Jilin University, Changchun 130025, PR China; 2 Department of Functional Materials Research, Central Iron and Steel Research Institute, Beijing 100081, PR China

## Abstract

The wettability of amorphous and annealing-induced nanocrystalline Fe_78_B_13_Si_9 _ribbons by molten Sn and Bi at 600 K was measured using an improved sessile drop method. The results demonstrate that the structural relaxation and crystallization in the amorphous substrates do not substantially change the wettability with molten Bi because of their invariable physical interaction, but remarkably deteriorate the wettability and interfacial bonding with molten Sn as a result of changing a chemical interaction to a physical one for the atoms at the interface.

## Introduction

Amorphous and nanocrystalline alloys are newly developed materials with a number of superior physical, chemical, and mechanical properties, which are of significant importance for basic scientific research and potential engineering applications [1,2]. The amorphous alloys are characterized by short-range order and long-range disorder, without the presence of any grain boundary in their crystallographic structure, whereas, the nanocrystalline materials possess high specific surface areas and a large density of grain boundaries or interphase boundaries. These distinct features are expected to bring about novel phenomena such as wetting at their surfaces/interfaces different from those of conventional coarse-grained polycrystalline substrates [3]. On the other hand, up to now, the maximum dimensions of the bulk amorphous and nanocrystalline alloys that can be directly prepared are still quite limited, typically no more than centimeters, thus constraining their applications in many situations. A potential way to achieve breakthrough in the size limitation is to develop appropriate joining techniques [4,5]. Regarding the fact that the structures of both the amorphous and nanocrystalline alloys are thermodynamically metastable, being very sensitive to heat treatment, the heat input in the joining should be carefully controlled to avoid a wide range of crystallization of the amorphous alloys and overgrowth of the nanocrystallites, which can deteriorate properties. Soldering, as a low-temperature joining process, is a potentially feasible method [5], and the wettability of these metastable alloys by molten solders plays a crucial role in this process [6]. However, only a few preliminary studies [6-10] have so far focused on this important issue, leaving many fundamental questions unsolved.

In the large family of the amorphous alloys, the Fe_78_B_13_Si_9 _alloy represents a basic metallic glass-forming material with clear crystallization kinetics [11-13]. The wetting behaviors of the amorphous and crystalline Fe_78_B_13_Si_9 _alloys by molten Sn and a low-melting-point Sn-57 wt%Bi alloy have been investigated by Zhang and colleagues [7-10] using a conventional sessile drop method. They reported that the equilibrium contact angles of liquid Sn or the Sn-Bi alloy on the amorphous Fe_78_B_13_Si_9 _substrates did not decrease monotonically with increasing temperature, and pre-annealing of the substrate deteriorated the wettability [7,8]. However, the explanations for the results are somewhat self-contradictory by the same group. For instance, Xu et al. [7] claimed that the surface energy (σ_sv_) of the amorphous alloy was smaller than that of the crystalline alloy, while Ma et al. [8,10] argued an opposite trend. Moreover, Xu et al. [7] suggested that the interfacial reaction for the formation of intermetallic compound (IMC) (they did not identify the nature of the product) should provide a driving force for the wetting of the amorphous substrate, while the structural relaxation and crystallization of the substrate impede the interfacial reaction and thus deteriorate the wettability. Instead, Ma et al. [8] proposed that the crystallization reaction should provide an additional force for the spreading. On the other hand, inconsistency also exists in the interfacial microstructures in Ma et al.'s study. In Ref. [9], they indicated the formation of a reaction (in fact, diffusion) layer at the interface of the amorphous substrate while no such layer at the interface of the crystalline substrate, whereas, in another article [10], they claimed that the width of the diffusion layer for the Sn-Bi alloy on the amorphous substrate was much thinner than that on the crystalline substrate. In addition, a puzzling question is that the initial contact angles reported by Ma et al. [8,10] are always 90°, irrespective of the experimental conditions. This result is rather questionable. We presume that they might have deliberately used the value of 90° as the initial contact angle. In fact, when using the conventional sessile drop method, in which the metal to be molten (e.g., Sn or the Sn-Bi alloy) was preplaced on the amorphous or pre-crystallized substrate surface, and then the couple was heated in a contact mode to the desired testing temperature, it is almost impossible for them to obtain the true initial contact angles and spreading kinetics at temperatures higher than the melting point of pure Sn or the Sn-Bi alloy since the wetting and melting begin simultaneously. On the other hand, the separation of the pre-annealing treatment and the wetting test could make the pre-crystallized substrates readily polluted and difficult to be handled because of their fragile nature.

In this article, we investigated the wettability of amorphous and annealing-induced nanocrystalline Fe_78_B_13_Si_9 _surfaces by molten pure Sn and Bi using an improved sessile drop method with a primary purpose to clarify the effect of this structural transition on the wettability as well as to determine the key factor that controls the wettability between them.

## Experimental procedure

The amorphous Fe_78_B_13_Si_9 _ribbons were about 30 μm in thickness and 20 mm in width. Thermal analysis using a differential scanning calorimeter (DSC, Netzsch STA 409 PC, Germany) at a heating rate of 20 K min^-1 ^in an argon atmosphere revealed a glass transition temperature (*T*_g_) of 680 K and a crystallization onset temperature (*T*_c_) of 798 K, followed by two successive crystallization exothermic peaks at 810 and 826 K, respectively. It should be mentioned, however, that the crystallization kinetics of this amorphous alloy is sensitive to compositions (mainly B and Si) [11], heat conditions such as heating rate [12,13], and impurities like carbon [13].

Before the wetting experiment, the surface of the Fe_78_B_13_Si_9 _ribbons was carefully polished using diamond pastes to average roughness of 30 ± 10 nm (*R*_a_) and then immersed in acetone for ultrasonic cleaning. The pure Bi (>99.99 wt%) and Sn (>99.999 wt%) samples were cut into small cubes weighing 120 ± 10 mg. An improved sessile drop method was adopted, i.e., the liquid drop was dispensed from a small hole (1 mm in diameter) at the bottom of an alumina tube (99.6 wt% purity) only when the preferred testing temperature (600 K) was reached. Detailed information can be found elsewhere [14]. The most significant advantage of this improved method lies in the separate locating of the substrate and the pure metal, and thus the Fe_78_B_13_Si_9 _amorphous ribbons, which were initially fixed on an alumina support and adjusted to a horizontal position, can be pre-annealed at different temperatures ranging between 600 and 1000 K for 10 min in a high vacuum (approx. 3 × 10^-4 ^Pa) to produce various structures and phases through relaxation and crystallization, and then cooled at 15 K min^-1 ^to the constant temperature of 600 K for the wetting test. Other advantages include mechanical removal of the oxide film covering the drop surface and elimination of the prior interaction between the drop and the substrate during heating, thus making the measurement of contact angle more accurate and reliable. As soon as the liquid was dropped and rested on the substrate, photos were taken using a high-resolution digital camera at a maximum speed of two frames per second under the illumination of 50-mW parallel laser beams with a diameter of 30 mm. The captured drop profiles were analyzed by an axisymmetric drop shape analysis program to calculate the contact angles. The substrate surfaces outside the drop were examined by X-ray diffraction (XRD, D/Max 2500PC, Japan) to identify the structure and phase changes from the amorphous to crystalline states. Selected solidified wetting couples were cross-sectioned and polished for interfacial microstructure observation using a scanning electron microscope (JSM 5310, Japan) coupled with an energy dispersive spectrometer (EDS, link-ISIS, Oxford, England).

## Results and discussion

**Figure 1:**
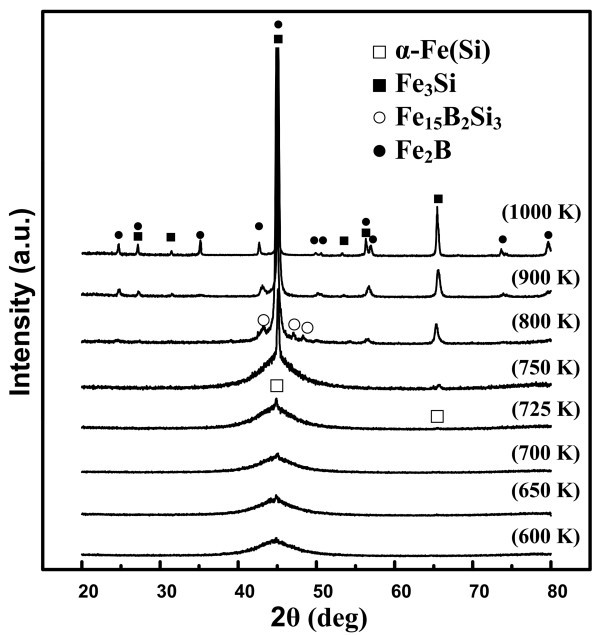
**XRD patterns for the Fe_78_B_13_Si_9 _substrates annealed at various temperatures for 10 min and then after the wetting at 600 K for 1 h**.

Figure 1 shows the XRD patterns of the Fe_78_B_13_Si_9 _ribbons after annealing at various temperatures for 10 min and then wetting at 600 K for 1 h. As indicated, when the annealing temperature (hereafter represented by *T*_a_) was no more than 725 K, the Fe_78_B_13_Si_9 _ribbons were basically still in the amorphous state. Nevertheless, as a result of heating, structural relaxation may take place, which caused changes in the arrangement of the atoms within the amorphous structure before the beginning of the crystallization. As the annealing temperature rose, a weak diffraction peak, corresponding to α-Fe(Si), began to appear when *T*_a _was 725 K, which was much lower than the crystallization onset temperature (*T*_c_) determined by DSC. This result might be explained by the fact that the thermal stability of the amorphous Fe_78_B_13_Si_9 _alloy is relatively poor and an isothermal dwell at temperatures lower than *T*_c _could lead to its crystallization [12,13]. With a further increase in *T*_a_, the amorphous structure gradually faded away, replaced by the emergence of multiphases such as α-Fe(Si), Fe_3_Si, Fe_15_B_2_Si_3_, and Fe_2_B in the matrix. However, only the Fe_3_Si and Fe_2_B IMCs were finally stable in the substrates annealed at high temperatures. Based on the Scherrer equation [15], the average grain size of the precipitated phases was estimated to be no more than 70 nm even after annealing at 1000 K for 10 min, suggesting that the ribbons were in the nanocrystalline state after polycrystallization.

**Figure 2:**
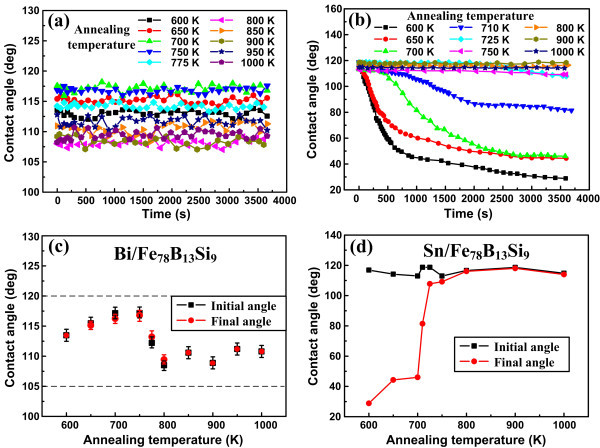
**Variations in contact angle with time at the constant wetting temperature of 600 K (*upper*) and variations in the initial and final contact angles with the substrate annealing temperature (*lower*)**. **(a, c) **for the Bi/Fe_78_B_13_Si_9 _system and **(b, d) **for the Sn/Fe_78_B_13_Si_9 _system.

Figure 2a, b shows the variations in contact angle with time during the isothermal (600 K) wetting tests of the Bi and Sn drops on the Fe_78_B_13_Si_9 _ribbons annealed at different temperatures, and Figure 2c, d show the variations in the initial (at t = 0 s) and final (after wetting for 1 h) contact angles with *T*_a_, respectively. For the Bi/Fe_78_B_13_Si_9 _system, the contact angles remained almost constant during the 1-h isothermal dwell and they did not vary noticeably with *T*_a_, even though a slightly larger value was observed at temperatures approaching the critical crystallization point (i.e., 725 K according to Figure 1a). Clearly, the Fe_78_B_13_Si_9 _ribbons could not be wetted by molten Bi, regardless of the annealing temperature, or more exactly, of the substrate structure and phase changes as well as grain growth. However, for the Sn/Fe_78_B_13_Si_9 _system, despite the fact that the initial contact angles do not vary considerably with *T*_a_, the final contact angles and the wetting dynamics indeed do, particularly for the substrates being in the apparently amorphous state (i.e., when *T*_a _is lower than 725 K). In this range, the contact angle decreased first rapidly and then progressively with time. The lower the annealing temperature, the faster the spreading rate, suggesting that structural relaxation in the amorphous substrates gives rise to an appreciable decrease in the wettability. As *T*_a _approached 725 K, the triple line of the liquid drop advanced very slowly and even stopped moving, indicating that the primary crystallization significantly deteriorates the wettability. After the crystallization, similar to that in the Bi/Fe_78_B_13_Si_9 _system, the contact angle did no longer change with time, nor with the annealing temperature. In this sense, the nanocrystalline Fe_78_B_13_Si_9 _substrates possess much poorer wettability by molten Sn compared with their amorphous counterparts.

**Figure 3:**
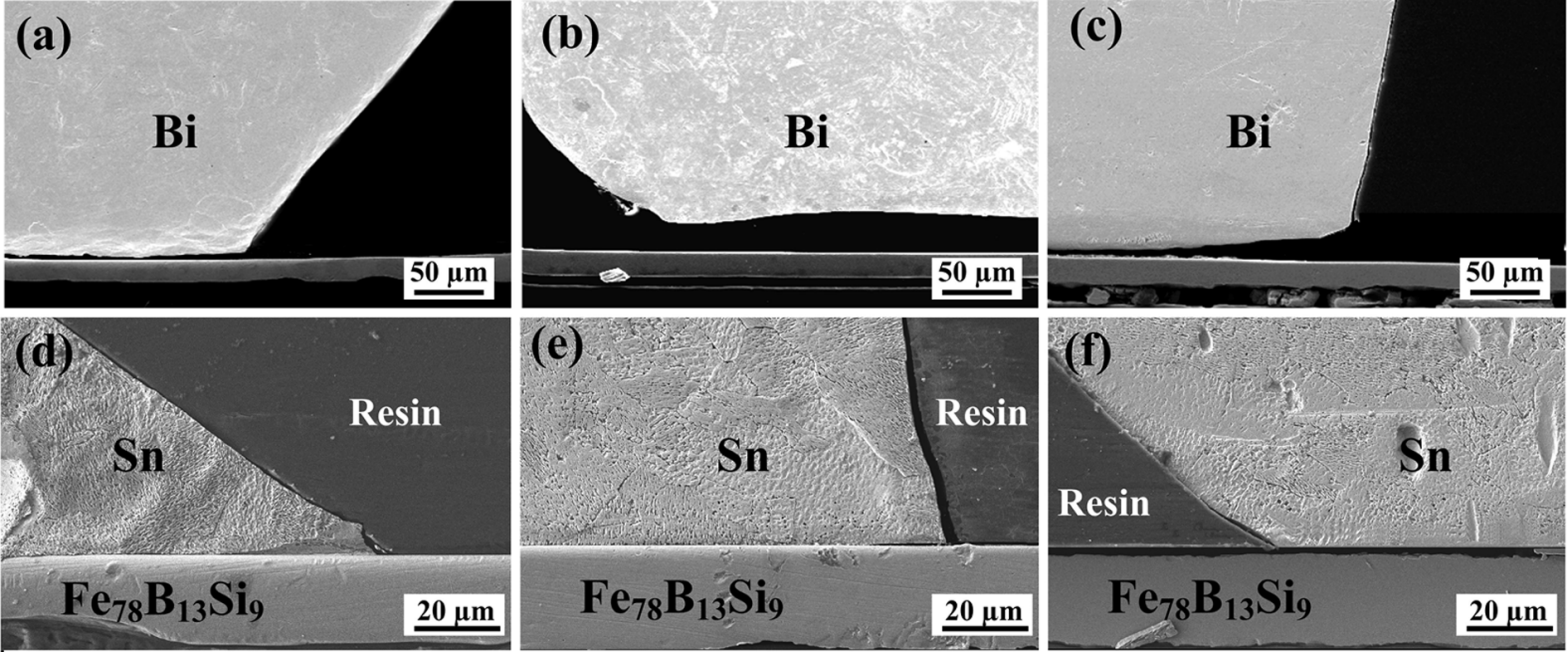
**Cross-sectional morphologies for the Bi and Sn drops on the Fe_78_B_13_Si_9 _ribbons annealed at various temperatures**. **(a, d) **600 K, **(b, f) **800 K, **(c) **1000 K, and **(e) **710 K.

Figure 3a, b, c shows the cross-sectional morphologies of the Bi-Fe_78_B_13_Si_9 _interfaces. All the Bi drops were separated from the Fe_78_B_13_Si_9 _surfaces during either cooling or later cutting for metallographic sample preparation, indicating very weak interfacial bonding. A thorough EDS analysis on both the cross-sectional interfaces and the separated surfaces (i.e., the bottom surface of the solidified drops and the contact surface of the substrates) revealed the absence of any reaction layer and diffusion layer. Figure 3d, e, f shows the cross-sectional microstructures of the Sn-Fe_78_B_13_Si_9 _interfaces. In contrast to the Bi-Fe_78_B_13_Si_9 _couples, a much more intimate contact was observed for the solidified Sn drops on the amorphous substrates, indicating strong interfacial bonding. However, for the as-crystallized substrates, separation was also observed, suggesting that the crystallization greatly weakens the interfacial bonding.

**Figure 4:**
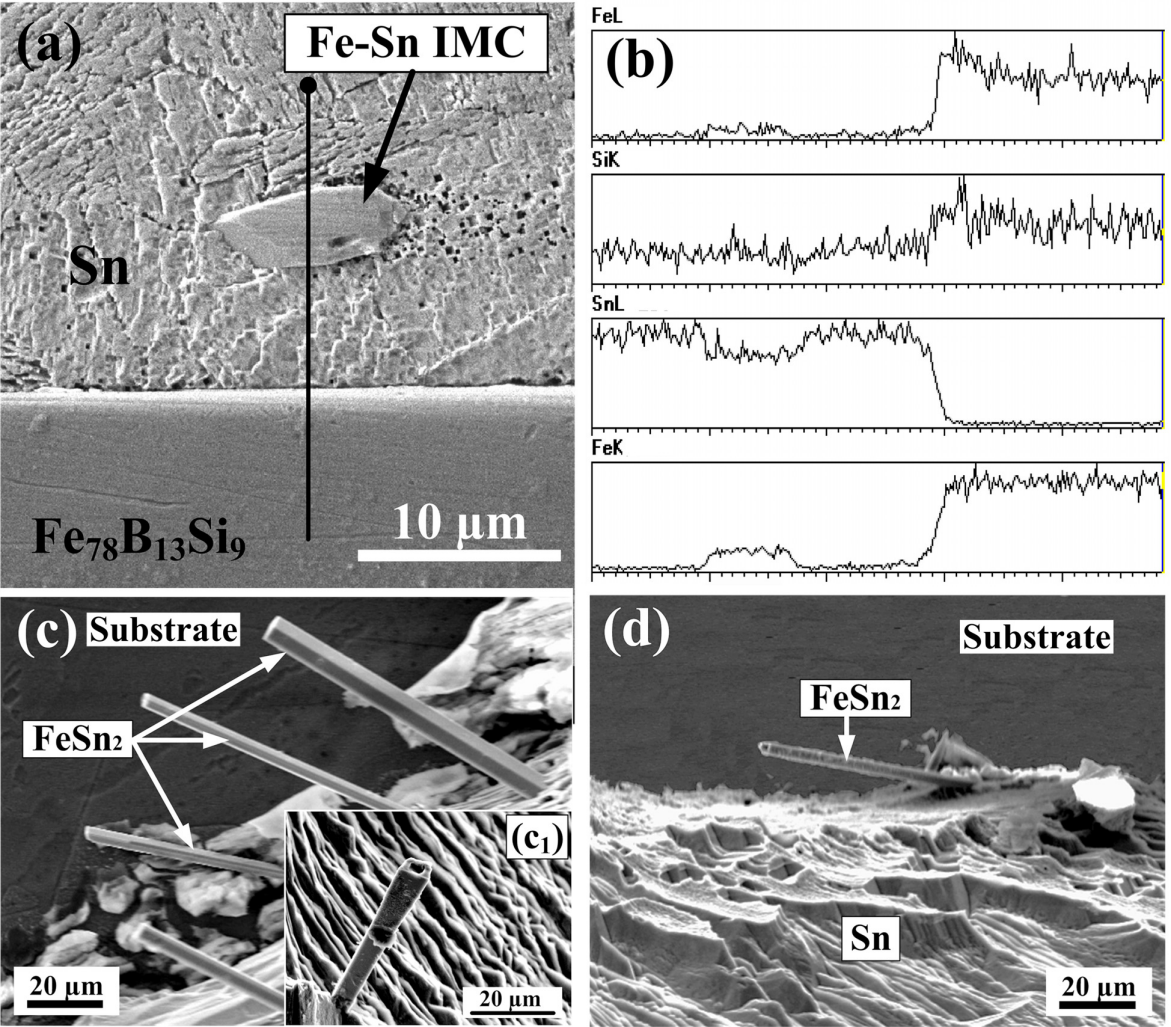
**Interfacial microstructure, compositional variation and morphology of the IMCs:** (a) Cross-sectional microstructure for Sn on the Fe_78_B_13_Si_9 _ribbon annealed at 600 K. **(b) **EDS analysis for the compositional change at the line position labeled in **(a)**. **(c, d) **Top-view morphologies of the exposed FeSn_2 _phase grown from the solid-liquid interface into the drop bulk for the substrates annealed at 600 K (**c**) and 700 K **(d)**, respectively, after partial removal of the solidified Sn drops. The inset in **(c) **shows the internal hollow structure of the FeSn_2 _phase.

Figure 4a shows the cross-sectional microstructure for Sn on the Fe_78_B_13_Si_9 _ribbon annealed at 600 K. The presence of an IMC phase was observed in the vicinity of the interface. After partial removal of the Sn drop in a 5%HNO_3_-3%HCl-92%ethanol (in volume) solution and viewed from the top surface, the IMC phase, in fact, nucleated at the interface and then grew into the Sn drop in the shape of bars or tubes, as shown in Figure 4c. The EDS analysis demonstrated that these IMCs were FeSn_2 _and there was no visible diffusion layer in the substrate (Figure 4b), which is quite different from the observation of Ma et al. [9,10], as we have described before. On the other hand, the amount of the FeSn_2 _phase decreased considerably with increasing *T*_a _(e.g., compare Figure 4c with 4d) and no such phase was found for the substrates annealed at temperatures higher than 710 K.

According to the Young equation,(1)

the equilibrium contact angle (θ_eq_) is determined by the difference in the solid surface free energy, *σ*_sg_, and the solid-liquid interfacial free energy, *σ*_sl_, providing that the liquid surface tension, *σ*_lg_, is a constant during the wetting. Again, based on a simple "nearest-neighbor" interaction model [16], *σ*_sg _and *σ*_sl _can be written as(2)(3)

where *ω *is the surface area per atom at the solid (s) surface or the solid-liquid (sl) interface, *Z *is the number of the nearest neighbors in the bulk crystal, *m *is the fraction of broken bonds at the surface of solid or liquid per atom, and ε_ss_, ε_sl_, and ε_ll _are the bond pair energies between solid-solid, solid-liquid, and liquid-liquid atoms, respectively. Assuming that the other parameters are constant, *σ_ij _*is then primarily dependent on ε*_ij_*. With the increase in *T*_a_, the volume of the Fe_78_B_13_Si_9 _ribbons underwent an appreciable change, which first expanded as *T*_a _approached the primary crystallization point and then decreased rapidly after crystallization, as clearly observed during the annealing treatment. Since the volume change reflects the variation in the average atomic bonding (ε_ss_) in the alloy, the solid surface free energy, *σ*_sg_, could be regarded as going through first a decrease and then an increase as *T*_a _increased from 600 to 1000 K, with the minimum value appearing at the primary crystallization point (725 K), where the volume expansion was the largest. On the other hand, an increase in ε_ss _usually corresponds to a decrease in ε_sl_. Accordingly, both *σ*_sg _and *σ*_sl _decreased and then increased with *T*_a_, as inferred from Equations 2 and 3. In other words, the effect of structural relaxation and crystallization on *σ*_sg _and *σ*_sl _seemed to more or less offset by the opposite changes in ε_ss _and ε_sl_, thus leading to a minor change in the initial contact angles for both the Bi-Fe_78_B_13_Si_9 _and Sn-Fe_78_B_13_Si_9 _systems as a function of the annealing temperature. A slightly larger contact angle for the substrates annealed at temperatures close to the primary crystallization point may suggest that the decrease in *σ*_sg _(or ε_ss_) should be more significant than that in *σ*_sl _as a result of enhanced volume expansion.

After the contact of the drop with the substrate, the subsequent wetting was then primarily dominated by the change in *σ*_sl_. For the Bi-Fe_78_B_13_Si_9 _system, the mixing enthalpies of Bi-Fe [17], Bi-B [18], and Bi-Si [19] are all positive, implying lacking of chemical affinity between Bi and the components in the Fe_78_B_13_Si_9 _alloy. Therefore, only physical interaction was developed at the interface and molten Bi cannot spread on the Fe_78_B_13_Si_9 _surfaces, regardless of the changes in their structures, phases and grain sizes as a function of *T*_a_. On the other hand, despite that Sn also failed to combine with B and Si [18,20], it could react with Fe when the Fe was in a free atomic state in the substrate,(4)

At 600 K, the change in standard Gibbs free energy, , is -7.219 kJ mol^-1 ^[21]. As is known, the amorphous alloys were produced by rapid quenching. Under this circumstance, the atoms in the amorphous alloy seemed to be suddenly frozen from the liquid and were in an overall free-of-organization state in their crystallographic structure [22]. When these atoms contacted liquid Sn, a chemical interaction developed between Fe and Sn at the interface, which substantially increased ε_sl _and thus decreased *σ*_sl_, leading to an immediate spreading of the Sn drop. With increasing *T*_a_, on the one hand, *σ*_sg _decreased as a result of structural relaxation in the amorphous substrate, and on the other, ε_sl _might be weakened and thus *σ*_sl _increased because of local thermally activated bonding between the atoms such as Fe-B and Fe-Si in the matrix, as witnessed by the decreasing amount of the FeSn_2 _phase formed at the interface (e.g., Figure 4d). As a consequence, the spreading rate decreased. When *T*_a _approached, or even higher than, the primary crystallization point (i.e., 725 K), the crystallization occurred and the IMCs such as Fe_3_Si, Fe_15_B_2_Si_3_, and Fe_2_B formed. Based on the Miedema theory, the enthalpy of the solution for the Fe-B (-143.56 kJ mol^-1^) and Fe-Si (-116.2 kJ mol^-1^) pairs is much more negative than that for the Fe-Sn pair (-4.4 kJ mol^-1^) [23], indicating a much stronger affinity between the components in the substrate itself. On the other hand, thermodynamic calculations for the following reactions(5)(6)

also indicated Δ*G*^0 ^> 0 at 600 K [24,25], suggesting that they are unable to take place. Therefore, it is reasonable to infer that after the primary crystallization of the substrate, the liquid Sn atoms failed to combine with Fe in the nucleated and grown nanocrystalline phases, thus leading to poor wettability and weak interfacial bonding.

## Conclusions

1. The structural relaxation and crystallization in the amorphous Fe_78_B_13_Si_9 _substrates do not significantly change the initial wettability with molten Bi and Sn.

2. The wettability and interfacial bonding of Fe_78_B_13_Si_9 _by molten Bi are always poor, regardless of the structural and phase changes in the substrate, because of invariable physical interaction for the atoms at the interface.

3. The wettability of amorphous Fe_78_B_13_Si_9 _by molten Sn is much better than that of the nanocrystalline substrate. The structural relaxation and primary crystallization in the amorphous substrate remarkably deteriorate the wettability and the interfacial bonding because of transition from a chemical interaction to a physical one in nature.

4. From the viewpoint of wettability, the amorphous Fe_78_B_13_Si_9 _substrate can be joined by lead-free Sn-base solders at a temperature much lower than the pre-crystallization point.

## Abbreviations

DSC: differential scanning calorimeter; EDS: energy dispersive spectrometer; IMC: intermetallic compound; XRD: X-ray diffraction.

## Competing interests

The authors declare that they have no competing interests.

## Authors' contributions

PS conceived of this study, analyzed the experimental results and drafted the manuscript. JXS and JY carried out the wetting experiments and performed microstructure and phase analyses. YQ prepared the Fe_78_B_13_Si_9 _metallic glass ribbons. QCJ participated in the design and coordination of this study. All authors read and approved the final manuscript.
